# Dynamically Tunable Pseudo-Enhancement-Load Inverters Based on High-Performance InAlZnO Thin-Film Transistors

**DOI:** 10.3390/nano16030153

**Published:** 2026-01-23

**Authors:** Hao Gu, Jingye Xie, Chuanlin Sun, Tingchen Yi, Yi Zhuo, Junchen Dong, Yudi Zhao, Kai Zhao

**Affiliations:** 1 School of Information & Communication Engineering, Beijing Information Science and Technology University, Beijing 100101, China; 2 School of Integrated Circuits, Peking University, Beijing 100871, China

**Keywords:** InAlZnO transistors, integrated circuits, inverter, voltage gain

## Abstract

Oxide transistors have attracted significant interest in the field of integrated circuits (ICs). Among various oxide semiconductors, InAlZnO (IAZO) stands out as a promising candidate due to its potential for high mobility and excellent stability. In this work, we fabricate high-performance IAZO transistors with a field-effect mobility of 56.60 cm^2^/V·s, a subthreshold swing of 82.59 mV/decade, an on-to-off current ratio over 10^7^, and a small threshold voltage shift of 0.09 V and −0.03 V under positive and negative bias stress, respectively. Based on these transistors, Pseudo-Enhancement-Load (PEL) inverters were constructed. An adjustable bias voltage (V_BIAS_) was also introduced as an additional control parameter, which allows for flexible control of the trade-off between circuit performance and power consumption. The resulting inverters achieve a balance between static and dynamic performance, exhibiting a voltage gain of 1.83 V/V and a relatively low power consumption of 2.58 × 10^−6^ W (V_BIAS_ = 1.0 V). Our work demonstrates the potential of IAZO transistor-based PEL inverters for high-performance, low-power oxide IC applications.

## 1. Introduction

Oxide transistors have attracted considerable attention in the field of integrated circuits (ICs) over the past decade owing to their excellent electrical performance, good uniformity over a large area, low fabrication cost, and back-end-of-line (BEOL) compatibility [1,2,3]. These properties have enabled a wide range of emerging IC applications, including flexible microprocessors, advanced sensors, neuromorphic computing, and capacitor-less dynamic random-access memory (DRAM) [4,5,6,7]. Consequently, the development of high-performance oxide-transistor circuits has become a critical direction for next-generation ICs.

Among various oxide semiconductors, InGaZnO (IGZO) transistors have become a research focus [8,9,10]. Chang et al. employed n-type amorphous IGZO thin-film transistors to develop a low-power emission pulse generation circuit for active-matrix organic light-emitting diode (AMOLED) displays [11], while Park et al. utilized IGZO transistors to construct a neuromorphic system for spiking neural networks [12]. However, a major limitation of IGZO transistors is the trade-off between mobility and reliability, as improvements in mobility often come at the expense of reliability [13,14,15,16]. Furthermore, their poor thermal stability may induce substantial threshold voltage shifts under high-temperature processing conditions [17].

To overcome these limitations, InAlZnO (IAZO) is considered a potential alternative to IGZO due to its favorable material properties [18,19,20,21]. The Al-O bond effectively suppresses defect formation, enabling a better balance between high mobility and excellent reliability [19]. Furthermore, the incorporation of Al is known to improve the stability of oxide semiconductors [22]. For the development of reliable integrated circuits, achieving a balance between high mobility and stability is critical. In this work, we utilize the favorable electrical properties of IAZO to fabricate high-performance transistors. Based on these transistors, we then construct and demonstrate their functionality in dynamically tunable logic circuits.

In previous work, our group developed a surface engineering method based on an Al modification layer to realize high-performance and highly stable IAZO transistors [23]. Although their device-level properties have been well demonstrated, their applicability in practical logic circuits remains insufficiently explored [24]. To address this gap, this work presents a systematic investigation of inverter circuits constructed from these IAZO transistors. The IAZO channel layer was prepared via RF sputtering, and Pseudo-Enhancement-Load (PEL) inverter circuits were subsequently fabricated. A key advantage of the PEL inverter is that its operation can be tuned by a bias voltage (V_BIAS_), providing an additional degree of freedom for post-fabrication performance optimization. Both the static (voltage gain, power consumption) and dynamic (frequency response, output voltage) characteristics of the IAZO-based PEL inverters are characterized. Our results are expected to provide an experimental basis for the design of oxide-transistor logic circuits, and promote their application in high-performance, low-power, and high-density integrated circuits.

## 2. Materials and Methods

**Figure 1:**
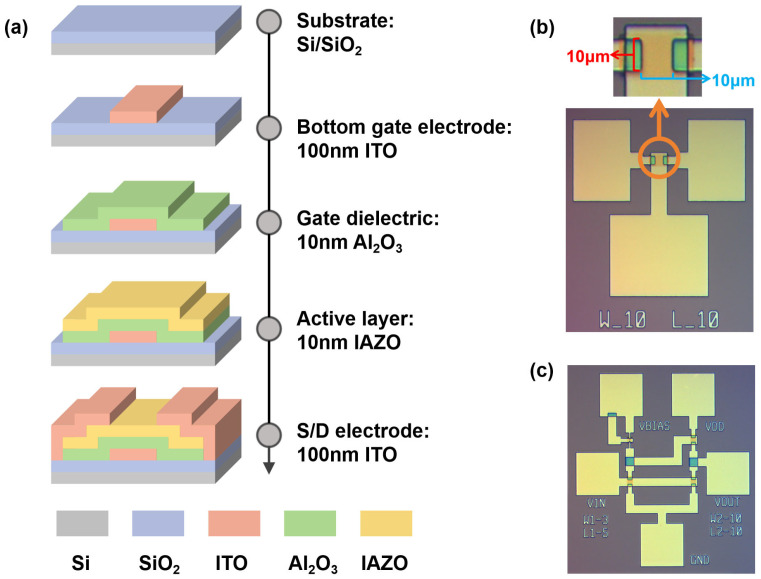
(**a**) Key process steps of IAZO TFT. (**b**) Layout of IAZO transistor. (**c**) Layout of PEL inverter based on IAZO transistors.

Figure 1a shows the fabrication process flow of the bottom-gate IAZO transistors. Firstly, p-type silicon wafers (0–0.0015 Ω·cm) with a 60 nm thermally grown SiO_2_ layer were pre-cleaned using an ultrasonic cleaner. Next, a 100 nm ITO film was deposited on the Si/SiO_2_ substrates as the gate electrode via radio frequency (RF) sputtering, followed by the deposition of a 10 nm Al_2_O_3_ gate dielectric via atomic layer deposition (ALD) at 120 °C. Subsequently, a 10 nm IAZO active layer was deposited via RF sputtering in an Ar/O_2_ mixed atmosphere at room temperature. A 100 nm ITO source/drain (S/D) electrode was then deposited by the sputtering process. Finally, the IAZO transistors underwent post-fabrication annealing at 400 °C in air for 1 h.

Figure 1b,c show optical photographs of the IAZO transistors and PEL inverters, respectively. The electrical properties of IAZO transistors and the static characteristics of inverters were measured using an Agilent B1500A semiconductor parameter analyzer, while the dynamic characteristics were measured using a waveform generator and an oscilloscope. During inverter testing, a supply voltage (V_DD_) of 1.0 V was applied, and the input voltage (V_IN_) was swept from −3 V to 3 V while monitoring the output voltage (V_OUT_) and the supply current (I_DD_).

## 3. Results

### 3.1. Properties of IAZO Films

**Figure 2:**
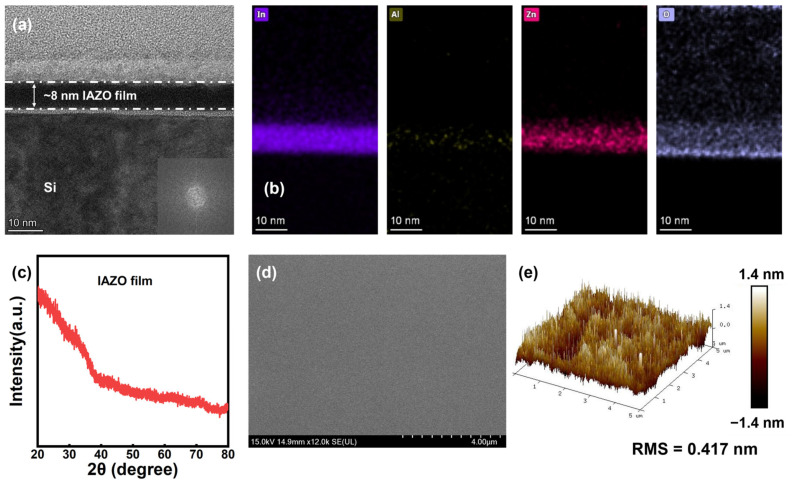
(**a**) TEM image and (**b**) EDS elemental mapping of a cross-section of IAZO film. (**c**) XRD pattern of IAZO film on the glass substrate. (**d**) SEM and (**e**) 3D AFM image of IAZO film on the Si substrate.

The microstructure of the IAZO film was characterized, as shown in Figure 2. Figure 2a presents a cross-sectional transmission electron microscopy (TEM) image of IAZO film deposited on a Si substrate. The film exhibits a uniform thickness of approximately 8 nm, with no evidence of localized crystallization. A fast Fourier transform (FFT) image of the film, shown in the inset of Figure 2a, further confirms its amorphous microstructure. Figure 2b presents the energy-dispersive spectroscopy (EDS) mappings of the IAZO film, showing that indium (In), aluminum (Al), zinc (Zn), and oxygen (O) elements are uniformly distributed throughout the film. X-ray diffraction (XRD) was performed on approximately 100 nm thick IAZO film grown on a glass substrate, as shown in Figure 2c. No distinct peaks were observed except for the substrate peak at ~25° [25]. This result indicates that the IAZO film is amorphous, which is consistent with the TEM analysis.

The surface morphology of the IAZO film was also examined using scanning electron microscopy (SEM) and atomic force microscopy (AFM). As shown in Figure 2d, the IAZO film exhibits a uniform and compact surface texture with well-distributed grains. The AFM image in Figure 2e, acquired over a 5 × 5 μm^2^ area, further reveals a very low root-mean-square (RMS) roughness of 0.417 nm. Both SEM and AFM results demonstrate that the fabricated IAZO film possesses a smooth surface, which can effectively reduce surface scattering and improve device performance [26].

### 3.2. Performance of IAZO Transistors

**Figure 3:**
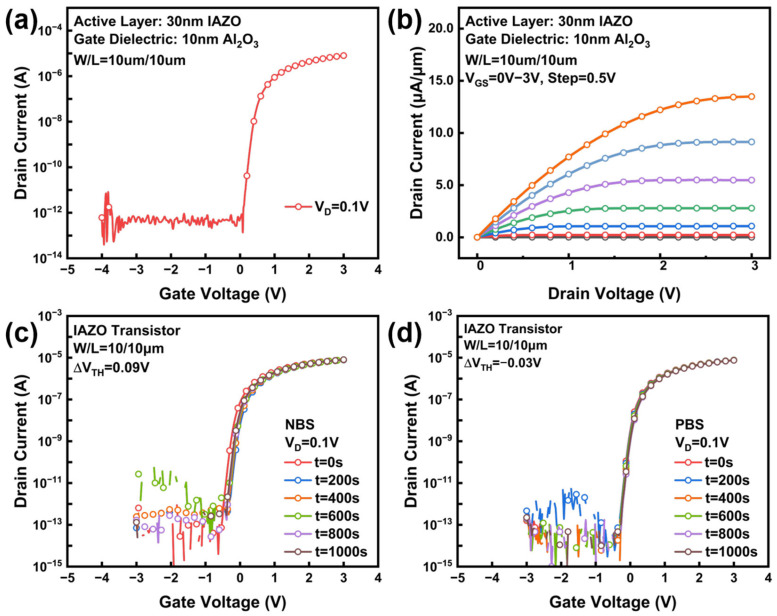
(**a**) Transfer curves of IAZO transistors. (**b**) Output curves of IAZO transistors. (**c**) NBS stability and (**d**) PBS stability of IAZO transistors. (Feature size is width/length (W/L) = 10 μm/10 μm. Drain voltage (V_D_) is 0.1 V.)

Figure 3a shows the drain current–gate voltage (I_D_-V_G_) curves of the IAZO transistors. The devices exhibit excellent transfer characteristics, with key electrical parameters including a field-effect mobility (µ_FE_) of 56.60 cm^2^/V·s, a subthreshold swing (SS) of 82.59 mV/decade, a turn-on voltage (V_ON_) of 0.08 V, and an on-to-off state current ratio (I_ON_/I_OFF_) over 10^7^. Figure 3b presents the output characteristics of the IAZO transistors. The output curves show clear linear and saturation regions, and no significant current crowding is observed in the linear region. Beyond the basic electrical performance, the operational stability under prolonged bias is crucial for practical circuit applications. Therefore, negative bias stress (NBS) and positive bias stress (PBS) tests were also conducted to evaluate the stability of the IAZO transistors. During the measurements, electric fields of −1 MV/cm and +1 MV/cm were applied to the gate dielectric for 1000 s under dark conditions. As shown in Figure 3c,d, the IAZO transistors demonstrate robust stability, with threshold voltage shifts (ΔV_TH_) of 0.09 V and −0.03 V under NBS and PBS, respectively. These results indicate that the IAZO transistors achieve a favorable balance between high performance and stability. A performance comparison with reported ZnO and IGZO TFTs is provided in Table 1.

### 3.3. Static Characteristics of Inverters

**Figure 4:**
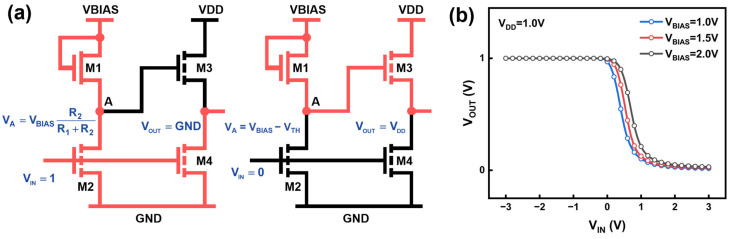
(**a**) Circuit schematic of PEL inverter (V_IN_ = 1 V, 0 V); (**b**) VTC of PEL inverters with varying V_BIAS_ (1.0 V–2.0 V).

Based on the IAZO transistors, we fabricated PEL inverters to explore their potential in logic circuits. As shown in Figure 4a, each PEL inverter consists of four transistors, with a width-to-length ratio (W/L) of 10/10 μm for M1 and 3/5 μm for M2, M3, and M4. This configuration ensures that the inverter functions correctly when a high-level voltage is applied to its input.

The operation of the PEL inverter can be divided into two phases [30]. When a high logic level is applied to the input, transistors M1, M2, and M4 are turned on simultaneously. The voltage at node A (V_A_) is determined by the voltage divider between M1 and M2. V_A_ must remain below threshold voltage (V_TH_) to turn off M3, allowing the output node to be rapidly pulled down to 0 V. When a low electrical level is applied, transistors M2 and M4 are turned off, and V_A_ equals V_BIAS_-V_TH_. To ensure that V_OUT_ reaches Voltage Drain-to-Drain (V_DD_), V_BIAS_ is typically set above V_DD_. As a result, M3 is turned on and drives the output node to V_DD_.

To quantitatively analyze the performance of the PEL inverter, the voltage gain and static power consumption are defined as follows: The voltage gain is defined as the maximum slope of the voltage transfer characteristic curve, representing its small-signal amplification capability:

(1) $$\mathrm{Voltage}\;\mathrm{gain}=\frac{{\mathrm{dV}}_{\mathrm{OUT}}}{{\mathrm{dV}}_{\mathrm{IN}}}$$

The static power consumption is determined by the quiescent current drawn from the supply and is calculated as

(2) $$\mathrm{Power}\;\mathrm{comsumption}=V_{DD}\cdot I_{DD}$$

where V_DD_ = 1.0 V and I_DD_ is the measured supply current.

**Figure 5:**
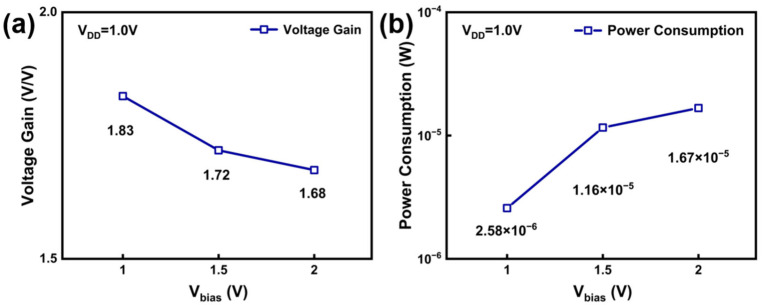
(**a**) Voltage gain and (**b**) power consumption of PEL inverters with varying V_BIAS_ (1.0 V–2.0 V).

A significant advantage of the PEL inverter is that both its voltage transfer characteristic (VTC) and power consumption can be tuned by the V_BIAS_. As shown in Figure 4b, increasing V_BIAS_ effectively changes the VTC of the PEL inverter. Notably, this improvement occurs without shifting the output levels, as output high voltage (V_OH_) and output low voltage (V_OL_) remain stable at 1.00 V and around 0.02 V, respectively. However, as V_BIAS_ increases, the voltage gain decreases from 1.83 V/V to 1.68 V/V as V_BIAS_ increases, as shown in Figure 5a. In addition, Figure 5b shows that when V_BIAS_ increases from 1.0 V to 2.0 V, the power consumption increases significantly, from 2.58 × 10^−6^ W to 1.67 × 10^−5^ W, nearly an order of magnitude. This increase is primarily attributed to the higher static current through the load transistor path (M1 and M2) under elevated V_BIAS_. These results indicate a clear trade-off between the performance and power efficiency in PEL inverters, which can be strategically optimized by tuning V_BIAS_ according to application requirements.

The achieved gain of 1.83 V/V at V_BIAS_ = 1.0 V is sufficient for logic inversion. However, it is lower than that of typical complementary or depletion-load oxide inverters (>10 V/V) [31]. This is characteristic of the PEL topology when optimized for low voltage and low static power [24]. Our design prioritizes tunability and low power over high gain. For applications requiring higher gain, different transistor ratios or topologies would be needed. The key advantage of this PEL inverter is its tunability, making it suitable for systems with varying performance needs.

### 3.4. Dynamic Characteristics of Inverters

**Figure 6:**
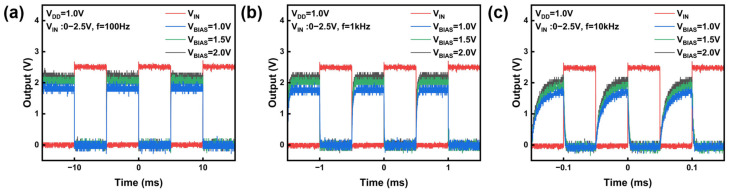
Output voltage waveforms of PEL inverter under square-wave input at different frequencies (100 Hz, 1 kHz, 10 kHz) and V_BIAS_ values: (**a**) 1.0 V, (**b**) 1.5 V, and (**c**) 2.0 V.

To further evaluate the application potential of IAZO inverters in digital circuits, we examined their dynamic response. A square-wave input signal with an amplitude of 2.5 V, a duty cycle of 50%, and frequencies of 100 Hz, 1 kHz, and 10 kHz was applied to the inverter. The corresponding output response was recorded using an oscilloscope. As shown in Figure 6, the output response gradually degrades with increasing frequency. This behavior is mainly caused by parasitic capacitances in the circuit. At higher frequencies, the available switching time becomes insufficient, and the charge/discharge rate can no longer follow the input transitions. Therefore, the output voltage swing decreases and waveform distortion becomes evident. This frequency-dependent performance degradation is a common challenge in ICs, primarily due to the limited charging and discharging rate of parasitic capacitance at the output node.

**Figure 7:**
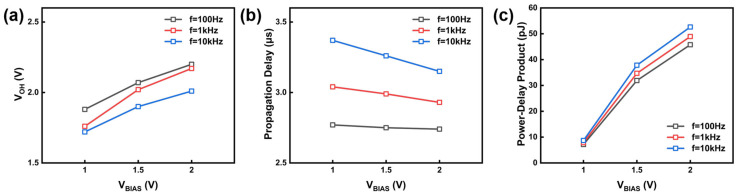
Dynamic characteristics of PEL inverter at different frequencies (100 Hz, 1 kHz, 10 kHz): (**a**) output high voltage; (**b**) propagation delay; (**c**) Power–Delay product.

Nevertheless, a key dynamic advantage of the PEL inverter is its ability to drive the V_OH_ close to V_IN_ (V_IN_ = 2.5 V), even under high-frequency operation. As depicted in Figure 6 and Figure 7a, increasing V_BIAS_ effectively enhances the pull-up capability of the inverter. This enhancement originates from the fact that V_BIAS_ influences the gate voltage of the pull-up transistor M3. When a low logic level is applied, the potential at node A becomes V_BIAS_ − V_TH_, providing a larger gate overdrive voltage for M3. As a result, M3 charges the output node more efficiently, allowing V_OH_ to approach V_DD_ even at higher operating frequencies. For instance, at 10 kHz, raising V_BIAS_ from 1.00 V to 2.00 V increases V_OH_ from 1.72 V to 2.01 V. This strong pull-up capability is crucial for preserving a high noise margin and signal integrity in multi-stage logic circuits.

In addition to output swing, the switching speed of the inverter was further evaluated through propagation-delay measurements. The propagation delay of the inverter is calculated based on the output waveforms shown in Figure 6. It is defined as the time difference between the input signal and the corresponding output signal:

(3) $$\mathrm{Delay}=\frac{\tau_{\mathrm{PLH}}+\tau_{\mathrm{PHL}}}{2}$$

where τ_PLH_ is the delay time when the output is switching from low to high and τ_PHL_ is the delay time when the output is switching from high to low. As shown in the input–output waveforms in Figure 7b, a higher V_BIAS_ leads to a shorter propagation delay. When V_BIAS_ increases from 1.0 V to 2.0 V, the delay decreases from 3.37 μs to 3.15 μs at 1 kHz. This improvement also stems from the enhanced drive strength of M3. A larger gate overdrive allows higher current to charge the output-node capacitance, accelerating the rising transition and thereby reducing the propagation delay.

The output waveforms at 10 kHz show a failure to reach full swing. This results from the limited charge/discharge rate of the output node. In our measurement setup, the dominant capacitive load arises from the probe pad capacitance and the gate–drain overlap capacitance of the transistors. To determine whether this limitation is intrinsic to IAZO, we estimated the transition frequency (f_T_) of a single transistor. The calculation is based on the standard formula

(4) $$f_{T}=\frac{g_{m}}{2\pi C_{gs}}$$

where g_m_ represents the transconductance and C_gs_ represents the gate-to-source capacitance. Using the measured peak g_m_ from Figure 3a, f_T_ is calculated to be in the range of hundreds of kHz to MHz. This value is significantly higher than the 10 kHz at which performance degradation occurs. Thus, the frequency limit is not from the IAZO material. It comes from external parasitics and large device size. Smaller devices and better layout will allow much faster switching.

To objectively evaluate the efficiency trade-off between static power consumption and switching speed offered by V_BIAS_ tunability, we calculated the Power–Delay product (PDP). The PDP is a key figure of merit for digital circuits, defined as

(5) PDP=Power·Delay

The calculated PDP values are shown in Figure 7c. As V_BIAS_ increases from 1.0 V to 2.0 V, the PDP increases. This shows that faster switching is achieved at the cost of a larger rise in static power. The minimum PDP of 7.15 × 10^−12^ J is achieved at V_BIAS_ = 1.0 V. Thus, V_BIAS_ serves as a tunable parameter for managing the speed–power trade-off. Depending on requirements, switching speed can be deliberately traded for improved power efficiency, or vice versa.

### 3.5. Comprehensive Analysis of Static and Dynamic Characteristics

The IAZO-based PEL inverter demonstrates excellent overall performance. In terms of static characteristics, it achieves high voltage gain and low static power while maintaining stable full-swing output. Dynamic tests further demonstrate good frequency response. Additionally, the propagation delay remains acceptable across the tested frequency range.

The key advantage of the PEL configuration lies in its tunability. By adjusting a single control variable, V_BIAS_, both static power consumption and dynamic performance can be regulated, providing designers with a direct method to optimize circuits. For high-speed applications, one can trade higher power for better performance. For power-sensitive uses, lower performance extends battery life. This flexibility meets diverse application needs. Compared to traditional inverters, the PEL structure maintains full-swing output while offering greater design flexibility, making it particularly suitable for dynamic power management and post-fabrication tuning. This tunability provides a practical and versatile solution for logic circuits in flexible and low-power electronics.

## 4. Conclusions

In this work, high-performance IAZO transistors were successfully fabricated and their potential in integrated circuits was demonstrated through PEL inverters. The IAZO transistors exhibit excellent electrical performance, including a high μ_FE_ of 56.60 cm^2^/V·s, a steep SS of 82.59 mV/decade, and good stability under both NBS and PBS. Based on these transistors, the PEL inverter exhibits a favorable balance between static and dynamic characteristics, maintaining a low static power consumption of 2.58 × 10^−6^ W while achieving a voltage gain of 1.83 V/V. A key advantage of this PEL inverter is its tunability via V_BIAS_, which provides design freedom to optimize the circuit after fabrication. By adjusting V_BIAS_, power consumption and dynamic performance can be effectively balanced. This work highlights the potential of IAZO-based circuits for future low-power and high-performance oxide electronics.

## Figures and Tables

**Table 1:** Comparison of key electrical parameters for ZnO, IGZO, and IAZO thin-film transistors.

References	[27]	[28]	[29]	This Work
Year	2024	2025	2025	2026
Active layer	ZnO	IGZO	IGZO	IAZO
μ_FE_ (cm^2^/V·s)	37.65	73.40	30.10	56.60
SS (mV/decade)	112.50	174.90	223.00	82.59
NBS (V)	−0.03	−0.20	−0.43	0.09
PBS (V)	−0.17	0.13	0.43	−0.03

## Data Availability

The data presented in this study are available on request from the corresponding authors.
